# Examining to what extent pregnancy-related physical symptoms worry women in the first trimester of pregnancy: a cross-sectional study in general practice

**DOI:** 10.3399/bjgpopen19X101674

**Published:** 2019-11-13

**Authors:** Melissa C Lutterodt, Pernille Kähler, Jakob Kragstrup, Dagny R Nicolaisdottir, Volkert Siersma, Ruth K Ertmann

**Affiliations:** 1 GP, The Research Unit for General Practice and Section for General Practice, Institute of Public Health, University of Copenhagen, Copenhagen, Denmark; 2 The Research Unit for General Practice and Section for General Practice, Institute of Public Health, University of Copenhagen, Copenhagen, Denmark; 3 GP, The Research Unit for General Practice and Section for General Practice, Institute of Public Health, University of Copenhagen, Copenhagen, Denmark; 4 The Research Unit for General Practice and Section for General Practice, Institute of Public Health, University of Copenhagen, Copenhagen, Denmark; 5 The Research Unit for General Practice and Section for General Practice, Institute of Public Health, University of Copenhagen, Copenhagen, Denmark; 6 GP, The Research Unit for General Practice and Section for General Practice, Institute of Public Health, University of Copenhagen, Copenhagen, Denmark

**Keywords:** general practice, first trimester of pregnancy, maternal health, signs and symptoms, primary health care

## Abstract

**Background:**

Women often wish to discuss their pregnancy symptoms with their GP. However, the two parties’ understanding of symptoms may not be aligned.

**Aim:**

To examine to what degree a specific pregnancy-related symptom worried women in the first trimester and analyse the characteristics of the most worried women.

**Design & setting:**

A cross-sectional study was performed in general practice in Denmark from 1 March 2015–15 August 2016.

**Method:**

Women attending the first prenatal care visit completed a questionnaire about pregnancy-related physical symptoms and worries. Women were recruited from 125 GP practices and 294 GPs participated in the study. Further data were obtained from their pregnancy health record. Multivariable logistic regression analysis was used to assess the associations between the women’s worries and the severity of the symptoms, which were adjusted for age and parity.

**Results:**

A total of 1508 women, aged 16–45 years, were included and 1455 completed the questionnaire. Nausea, vomiting, pelvic cavity pain, and back pain were the most common symptoms, and 88% reported having two or more symptoms simultaneously. Among the 1278 women reporting nausea, only 21% were worried, while 88% of the 252 women reporting vaginal bleeding were worried. Primigravidae (those pregnant for the first time) were significantly more worried about vomiting and nausea than multigravidae (those who have experienced pregnancy previously). Those aged >35 years were more worried about pelvic girdle pain and pelvic cavity pain than younger women.

**Conclusion:**

Pregnancy-related physical symptoms are frequent in the first trimester. The severity of worries depends on the symptom. Vaginal bleeding and pain give rise to the majority of severe worries, especially among young women.

## How this fits in

Pregnancy-related physical symptoms are common throughout pregnancy, and it is well known that women wish to discuss these symptoms with their GP. This study showed that in the first trimester, the majority of women already have more than one symptom. Most symptoms do not give rise to major concern, but symptoms involving pain or bleeding are worrisome to many women, and nausea is a frequent symptom causing minor worries among one-fifth of the women. Worries about symptoms should, therefore, be addressed explicitly by the caregiver at all prenatal care visits.

## Introduction

Up to 85% of women who are pregnant want to discuss pregnancy-related symptoms at the preventive health checks performed by their GP or the midwife.^1^ In Denmark the GP plays a central role in prenatal care services, in collaboration with midwives and obstetric departments. By Danish law, a minimum of three prenatal care visits are offered by the GP during pregnancy at weeks 6–10, 25, and 32. At the prenatal care visits, the focus is on identifying high-risk pregnancies and pregnancy complications.^2^ The pregnancy health records, therefore, support a biomedical focus on major health problems, and less attention is given to symptoms, such as nausea, that may be present in otherwise normal pregnancies.

Pregnancy-related physical symptoms are common in the first trimester.^1,3,4^ Nausea is reported by approximately 80% of women, while vomiting affects 35%–40% in the first trimester.^5–10^ Frequencies in the order of 25%–50% have also been described for symptoms such as vulvar itching, vaginal bleeding, and pelvic cavity pain.^11–13^ Pelvic girdle pain and low back pain (or, in some studies, a combination of these two symptoms referred to as ‘lumbo-pelvic pain’) are often reported for all three trimesters, with a prevalence of 20%–65%.^14–17^ While some symptoms might be pathological,^18–20^ most will be signs of a healthy pregnancy and be caused by physiological changes in the women’s endocrine system and physical status.^21^

There is sparse knowledge about how women perceive the threat from these symptoms, and GPs may be unaware of women’s worries in relation to physiological signs.^22^ Only a few studies have focused on women’s worries in relation to their physical symptoms during pregnancy.^1,23^ Various depression and anxiety scales, such as the Cambridge Worry Scale, have been used to assess maternal stress or worries during pregnancy, but these scales are global measures and do not address the impact of specific symptoms.^24–28^

The purpose of the present study was to examine to what degree each physical pregnancy-related symptom worried Danish women during the first trimester of pregnancy. Furthermore, the characteristics of the women who were the most worried were analysed.

## Method

### Study design

A cross-sectional study^29^ was undertaken in Danish general practices, comprising data on pregnancy-related physical symptoms among women in the first trimester of pregnancy. Data were obtained in relation to the first prenatal care visit.

### Setting

The healthcare system in Denmark is tax-funded and most care is free of charge for the patient. The majority of Danes are registered with a GP, who functions as gatekeeper to secondary care (such as hospitals). GPs work either single-handed or in partnerships with two or more doctors. The GP plays a central role in prenatal care services, which are shared between the GP, midwives, and obstetric departments. By Danish law, a minimum of three prenatal care visits are offered by the GP for pregnancy at weeks 6–10, 25, and 32. A fourth postnatal care visit is conducted 8 weeks after delivery. The first visit, attended by almost all pregnant women, precedes other contacts with the healthcare system. In this consultation, a thorough and structured record is established (the pregnancy health record), which is then sent to midwives and the hospital department. Four to six consultations with the midwife and one to two ultrasound screenings for foetal abnormalities are, furthermore, offered during pregnancy.

### Participants and procedure

GPs were recruited from two of the five Danish administrative regions (the Capital Region and Region Zealand), corresponding to a population of approximately 2.4 million, with a total of 1561 general practices organised in 53 geographic units. Nineteen of these geographic units were randomly selected for the study, and all GPs in these units were invited to participate in the study. Among the invited general practices, 192 accepted the invitation. Patients were successfully recruited from 125 of these practices (representing a total of 294 GPs). GPs were asked to consecutively include all pregnant women at the first prenatal care visit during the study period.

All pregnant women booking an appointment for the first prenatal care visit during the period 1 March 2015–15 August 2016 were eligible for inclusion in the project and were included after signing a consent form. Women were excluded if they did not complete the electronic questionnaires (all in Danish), if they withdrew consent, or if the pregnancy ended in abortion.

### Data collection

Data were collected from two sources: (1) an electronic patient questionnaire; and (2) the pregnancy health record (a mandatory record produced by the Danish health authorities). The pregnancy health record was filled in by the GP at the first prenatal care visit and a copy was sent to the administration of the project. The women received a link to the electronic questionnaire (SurveyXact) by email after the first prenatal care visit. It was only possible to return a completely answered questionnaire. Non-responders were sent the questionnaire again and, if they still did not respond, they received an email and text message (some were contacted by phone).

### Questionnaire

The pregnancy-related physical symptoms recorded in the questionnaire were chosen after thorough study of relevant literature and based on experience from general practice. Frequent physical symptoms with an expected potential to cause worry were chosen: nausea, vomiting, back pain, pelvic girdle pain, pelvic cavity pain, vulvar itching, varicose veins, pruritus, leg cramp, and vaginal bleeding.

For each symptom (x) the questionnaire contained the following question: During this pregnancy, have you so far had 'symptom x'? Symptoms were to be categorised as ‘not present*’* *,* ‘mild’*,* ‘moderate’ or ‘severe’*,* except for the symptom 'vaginal bleeding', which was to be categorised as either ‘not present’ or ‘present’. The questions concerning back pain and pelvic girdle pain were explained by illustrations of the spine and pelvis with arrows pointing out the areas of interest.

In relation to all symptoms the women were further asked: Have you been worried about 'symptom x'? These answers were categorised as ‘no’*,* ‘mildly’*,* ‘moderately’, or ‘severely’. The women’s answers depended on their own subjective experience or perception of a given symptom.

### The pregnancy health record

Information from the pregnancy health record was used regarding age (categorised as 16–25 years, 26–35 years, and 36–45 years) and parity (categorised as primigravida [first pregnancy] or multigravida [having had more than one pregnancy]).

### Statistical analysis

Pearson’s correlation coefficient (*r*) was used to investigate the co-occurrence of symptoms (data collected from the questionnaire). The associations between worries and degree of physical symptom, age, and parity were assessed for each symptom separately by multivariable logistic regression analysis limited to those women presenting that symptom (data collected from the questionnaire and the pregnancy health record). The statistical analyses were performed with SAS (version 9.4) and R (version 3.3.1). A *P* value <0.05 was considered statistically significant.

## Results

A total of 1508 women gave informed consent to participate. Among these women, 29 were excluded owing to spontaneous abortion, and 24 did not return the questionnaire. Data from 1455 women were, therefore, available for analysis regarding the prevalence of symptoms and worries of the women.

The prevalence of early pregnancy-related physical symptoms among the 1455 participating women is presented in Table 1. Nausea was experienced by 88% of women, while vomiting, pelvic cavity pain, pelvic girdle pain, and back pain were reported by 34%–56%. The remaining pregnancy-related physical symptoms were each experienced by <20% of the women. Vaginal bleeding was experienced by 17%.

**Table 1.** The prevalence of self-reported pregnancy-related physical symptoms, classified by severity among 1455 women in the first trimester

	**Symptom not present, *n* (%**)	**Mild, *n* (%**)	**Moderate, *n* (%**)	**Severe, *n* (%**)
**Nausea**	177 (12)	503 (35)	379 (26)	396 (27)
**Vomiting**	876 (60)	325 (23)	134 (9)	120 (8)
**Back pain**	907 (62)	390 (27)	99 (7)	59 (4)
**Pelvic girdle pain**	967 (66)	357 (25)	84 (6)	47 (3)
**Pelvic cavity pain**	636 (44)	660 (45)	118 (8)	41 (3)
**Vulvar itching**	1182 (81)	206 (14)	42 (3)	25 (2)
**Varicose veins**	1415 (97)	30 (2)	7 (1)	3 (0)
**Pruritus**	1210 (83)	200 (14)	28 (2)	17 (1)
**Leg cramp**	1309 (90)	118 (8)	15 (1)	13 (1)

The majority of women (88%) experienced two or more symptoms during the first trimester, and only 2% had no symptoms. A statistically significant co-occurrence was observed between vomiting and nausea (*r* = 0.29); back pain and pelvic girdle pain (*r* = 0.33); pelvic girdle pain and pelvic cavity pain (*r* = 0.24); and, furthermore, between back pain and pelvic cavity pain (*r* = 0.14). Less strong, but statistically significant, correlations were found between a number of the remaining symptoms (*r* = 0.06–0.12).

**Figure 1.**
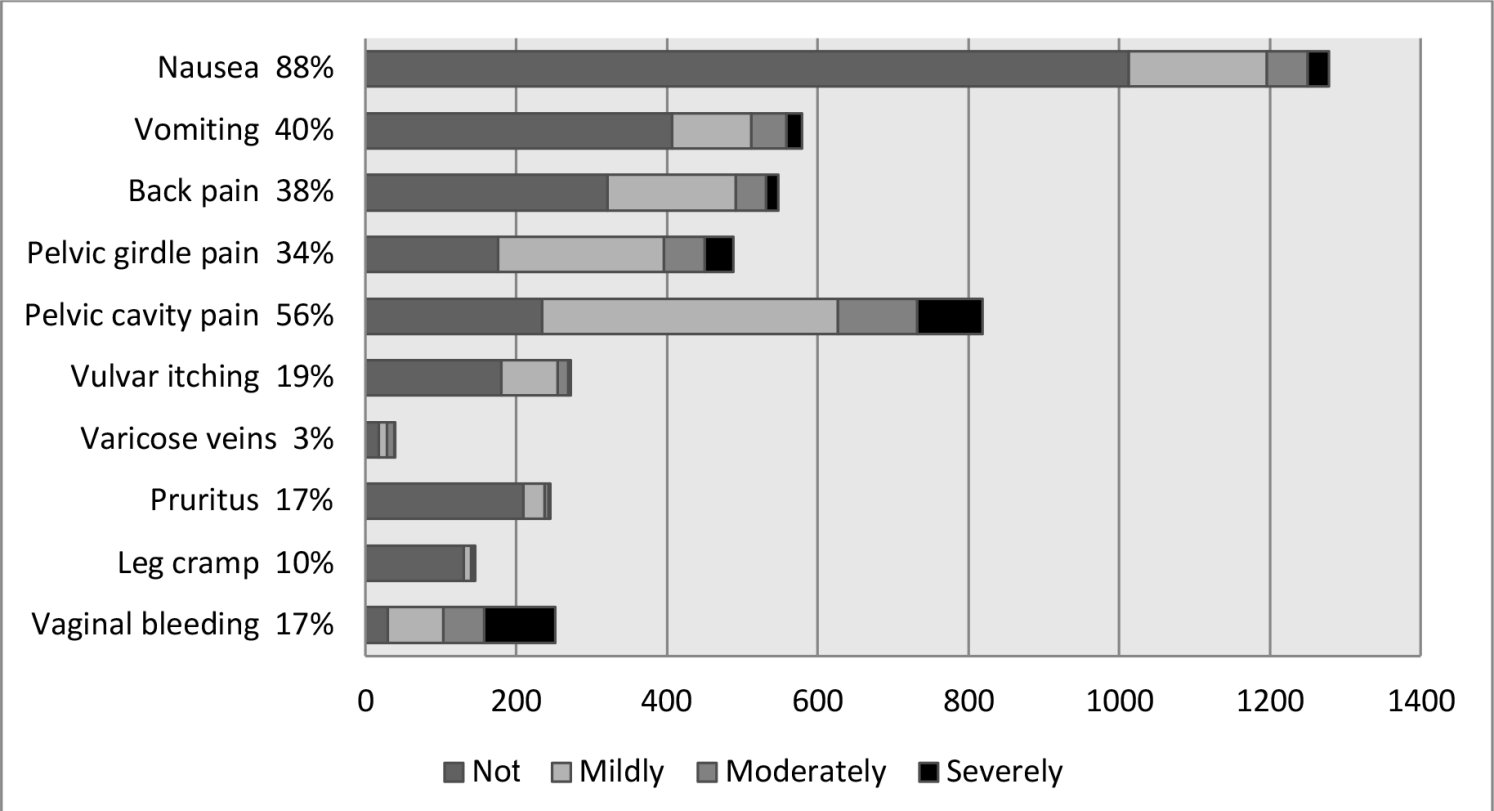
Prevalence and degree of worry for each pregnancy-related physical symptom. Each row illustrates the prevalence of a symptom (any degree). The number of women worried by each symptom is illustrated by the different shading (not, mildly, moderately, and severely).

As illustrated in Figure 1, not all symptoms gave rise to worries, but some symptoms were worrisome to a higher degree. Among the 1278 women reporting nausea, 265 (21%) were worried (most to a mild degree), while 222 (88%) of the 252 women reporting vaginal bleeding were worried. Pelvic cavity pain also gave rise to worries (71% of the 819 women), but the majority only reported mild or moderate worries.

The pregnancy health records were not obtained for 13 women and thus full datasets (that included parity) for the multivariable analysis, were available for 1442 women. Strong associations were found (odds ratio [OR] 3.1–21.6) between the severity of the pregnancy-related physical symptoms (‘mild’ versus ‘moderate and severe’) and the worries they raised (Table 2). The association with severity was strongest for vomiting (OR 14.7) and pruritus (OR 21.6). Women aged 36–45 years were significantly less worried about vulvar itching (OR 0.3) than younger women. Finally, multigravidae reported being significantly less worried about vomiting (OR 0.6) and nausea (OR 0.7) than primigravidae.

**Table 2.** Associations between worries and degree of physical symptom, age, and parity

Symptom	**Prevalence,** *n* (%)	Worry, *n*	Association between degree of physical symptom and worriesOR (95% CI)	Association between age in years and worriesOR (95% CI)	Association between parity and worriesOR (95% CI)
			**Mild**	**Moderate or severe**	**16** **–** **25**	**26** **–** **35**	**36** **–** **45**	**Primigravida**	**Multigravida**
Nausea	1269 (88)	264	1.00	5.0 (1.6 to 16.2)^a^	1.0 (0.7 to 1.5)	1.00	0.9 (0.7 to 1.3)	1.00	0.7 (0.5 to 0.9)^a^
Vomiting	576 (40)	171	1.00	14.7 (4.6 to 47.1)^a^	1.0 (0.6 to 1.6)	1.00	0.7 (0.4 to 1.2)	1.00	0.6 (0.4 to 0.8)^a^
Back pain	542 (38)	224	1.00	5.5 (2.4 to 12.4)^a^	0.8 (0.5 to 1.3)	1.00	1.1 (0.7 to 1.8)	1.00	1.1 (0.8 to 1.6)
Pelvic girdle pain	482 (33)	308	1.00	5.3 (3.2 to 8.7)^a^	1.0 (0.6 to 1.7)	1.00	1.4 (0.8 to 2.3)	1.00	0.8 (0.6 to 1.2)
Pelvic cavity pain	812 (56)	579	1.00	5.0 (3.6 to 7.0)^a^	1.2 (0.7 to 1.8)	1.00	1.3 (0.9 to 2.1)	1.00	0.8 (0.6 to 1.0)
Vulvar itching	267 (19)	90	1.00	3.1 (1.3 to 7.3)^a^	0.9 (0.5 to 1.9)	1.00	0.3 (0.1 to 0.7)^a^	1.00	0.7 (0.4 to 1.2)
Varicose veins	39 (3)	22	1.00	3.8 (1.0 to 14.6)^a^	( - )	1.00	3.7 (0.7 to 20.8)	1.00	2.6 (0.5 to 13.1)
Pruritus	241 (17)	35	1.00	21.6 (2.9 to 160.8)^a^	0.5 (0.2 to 1.5)	1.00	0.9 (0.3 to 2.4)	1.00	1.2 (0.6 to 2.4)
Leg cramp	144 (10)	15	( - )	( - )	0.5 (0.06 to 4.2)	1.00	2.1 (0.7 to 6.5)	1.00	1.4 (0.5 to 4.5)

CI = confidence intervals. OR = odds ratio.

The associations between worries and degree of physical symptom (mild or moderate or severe), age (16–23 or 26–35 or 36–45) and parity (primigravida or multigravida). Associations are assessed for each symptom separately as odds ratios obtained from a multivariable logistic regression model performed among the women presenting that symptom.

^a^Statistically significant results (*P*<0.05).

## Discussion

### Summary

Nausea was the most frequent symptom, but symptoms such as pelvic cavity pain, pelvic girdle pain, and back pain were also frequent in the first trimester of pregnancy. The majority of women (88%) experienced two or more symptoms during the first trimester. Many symptoms, such as nausea, vomiting, and pruritus, did not cause worry in the majority of women, but pain and bleeding often gave rise to worries. Worries were more common among young women and among those pregnant for the first time.

### Strengths and limitations

The group of pregnant women included in the present survey was relatively large compared with previous studies and can be regarded as representative of Danish pregnant women. No exclusion criteria were applied; although, a small group of women who did not speak Danish was less likely to be represented, and thus results might be less applicable to these women. It is possible that not all eligible women were asked by the GPs to participate in the study; however, the GPs were not given any information about the focus of the present article, and it is unlikely that any selection is related to pregnancy complaints. The general practices that voluntarily chose to contribute constitute approximately 40% of those asked. However, the participating GPs were sampled by a systematic procedure based on random selection from two regions of Denmark, including urban and rural areas, and areas with low as well as high social status. This strengthens the sociodemographic and geographical representation of the study. No standard instrument existed for the questionnaire about physical symptoms in pregnancy. The questionnaire used in this study has not been validated against a 'gold standard' and, therefore, it is not known exactly how the women comprehend, for example, 'nausea'. Symptoms such as 'nausea' or 'leg cramp' may have good face validity, while the women's understanding of symptoms such as 'pelvic girdle pain' and 'back pain' may be more uncertain. Therefore, the validity of the answers for 'pelvic girdle pain' and 'back pain' were improved by using an illustration of the areas of interest. There were very few non-responders among women who had enrolled in the study, and the complete datasets obtained from almost all participants furthermore reinforce the representativeness and external validity.

### Comparison with existing literature

The data regarding prevalence of pregnancy-related physical symptoms are in good accordance with those reported globally and in recent Scandinavian studies.^6,15,16^ The associations between symptoms found in the present study are also in good accordance with other reports.^5–10,14,15^ It was found that 88% of the women, including both primigravidae and multigravidae, reported more than two pregnancy-related physical symptoms. An Australian study including only primigravidae reported that 68% had three or more health issues occasionally or often.^17^ The health issues included were: exhaustion, frequent coughs and colds, migraines, constipation, haemorrhoids, depression, anxiety, relationship problems, morning sickness, vaginal bleeding, pelvic pain, and back pain. Their population and the included symptoms are, therefore, not directly comparable with the group of women in the present study, and the present authors are not aware of other studies with a focus on the number of symptoms reported.

The present study contributes new knowledge regarding the worries found in early pregnancy, especially with regards pain such as back pain, pelvic girdle pain, and pelvic cavity pain. Half the women with these symptoms were worried, and such worries might affect the further progression of the pregnancy.^13,30^ The large Norwegian Mother and Child Cohort showed that severe pelvic girdle pain combined with emotional distress was associated with non-recovery after delivery.^30^ Furthermore, pain-related anxiety in pregnancy has been shown to be associated with greater pain intensity and unpleasantness of postpartum genital-pelvic pain.^13^ It is possible that some of these women could be helped earlier and maybe not suffer from lack of recovery from pelvic girdle pain postpartum, if addressed at the first prenatal care visit.

To the authors' knowledge, no existing studies have reported the severity of the women’s worries in relation to vaginal bleeding. Given that 88% of the women were worried because of vaginal bleeding (and the percentage may be even higher considering the 29 women with spontaneous abortions), this calls for GPs’ attention to this specific group of patients during the first trimester.

Since nausea and vomiting are frequent physical symptoms in early pregnancy (88% and 40%), women being worried to any degree about these symptoms cover a large number of the pregnant women attending the first prenatal care visit. Knowing that 85% of women want to discuss pregnancy-related symptoms,^1^ it is, therefore, important that the GP pays special attention to asking women about these two symptoms at the first prenatal care visit. Furthermore, it is suggested that attention should be focused especially on primigravidae, considering that the present study shows that they worried a great deal more than multigravidae with regard to vomiting and nausea.

### Implications for practice

Worries about symptoms should be addressed explicitly by the caregiver at all prenatal care visits. It has previously been shown that most women want to discuss pregnancy-related symptoms with their GP, and this study shows that a large fraction of women already have one or more symptoms that worry them in the first trimester. Special attention should be given to symptoms involving pain or bleeding, but even a symptom like nausea may produce some level of concern among one-fifth of the pregnant women.
